# Supplementary material for: Comparison of the predictive ability for perinatal acidemia in neonates between the NICHD 3-tier FHR system combined with clinical risk factors and the fetal reserve index
Source: PLoS One. 2022 Oct 20;17(10):e0276451. doi: 10.1371/journal.pone.0276451 (PMC9584503; doi:10.1371/journal.pone.0276451)
Supplement: Attachment — Submitted filename: Response to Reviewers.docx [file pone.0276451.s001.docx]

**Review Comments to the Author**

Please use the space provided to explain your answers to the questions above. You may also include additional comments for the author, including concerns about dual publication, research ethics, or publication ethics. (Please upload your review as an attachment if it exceeds 20,000 characters)

Reviewer #1: The study reports the prediction of asphyxia (?) using a combination of FHR evaluation and risk factors. The approach proposed try to objectify the well-known clinical effort of consider multiple factors and not only the fetal heart rate during labor to bring decision on delivery.
Some methodical limits should be considered.

1. **The number of cases is small. The choice of the sample is not clear.**

The authors explained additional information about the required study population size.

In revised version:

Materials and method (Page 4, line 75-80): “The required study population size was determined based on comparison of area under the curve (AUC) of receiver operating characteristic (ROC) curves between the NICHD 3-tier system alone (estimated at 0.55) and the NICHD 3-tier system combined with risk factors or the FRI (estimated at 0.70) with approximately 12% of neonates having acidemia. For power of 80% and alpha of 0.05, and allowing for up to 20% incompleted data, 658 were required [16]”

**2) Why did the authors exclude infants with no umbilical cord gas results? The diagnosis of “perinatal asphyxia” may be made even in their absence.**

Birth asphyxia or cerebral palsy may occur in antepartum or intrapartum period. Distal risk factors (genetic abnormalities, environmental/sociodemographic factors, and some placental abnormalities) often exert a pathogenic effect on fetal brain starting at conception or antepartum period. While, proximal risk factors (such as abruptio placenta, TTTS, chorioamnionitis) often exert a pathogenic effect on fetal brain at intrapartum period.

The authors focused only at intrapartum period. Perinatal acidemia implied that fetuses had intrapartum hypoxic component. However, in revised version, the authors changed the term of asphyxia to acidemia, as the reviewer’s suggestion.

2. **The authors should define or justify indication for continuous fetal heart rate monitoring, UA pH determination, choice of cut-off for asphyxia.**
1. In our center, continuous fetal monitoring was implemented in all pregnancies.
2. Umbilical cord blood gas was collected in pregnancy who had the NICHD 3-tier FHR category I with maternal, obstetric, or fetal risk factors, the NICHD 3-tier FHR category II and III.
3. The authors defined perinatal acidemia as pH < 7.2 or base deficit ≥ 12 mmol/L according to reference [4] that define as if the cord gas pH level ≥ 7.2, it is **unlikely** that peripartum played a role in causing neonatal encephalopathy
3. **Is the sample a cohort or a convenience one? More information on the suggested or adopted guidelines for FHR interpretation in the center of the study, used to decide the timing of delivery, should be clarified.**
1. This was a retrospective cohort study that recruited a total of 674 pregnant women (80 acidemia, 594 non-acidemia). The authors have more explained information about the required study population size.

In revised version: Materials and method (Page 4, line 75-80):

“The required study population size was determined based on comparison of area under the curve (AUC) of receiver operating characteristic (ROC) curves between the NICHD 3-tier system alone (estimated at 0.55) and the NICHD 3-tier system combined with risk factors or the FRI (estimated at 0.70) with approximately 12% of neonates having acidemia. For power of 80% and alpha of 0.05, and allowing for up to 20% incompleted data, 658 were required [16]”

2. The authors explained additional information about adopted guideline and interpretation

In revised version: Material and methods (Page 4, line 73-74): “The FHR tracings were interpreted and managed according to the NICHD 3-tier FHR system [1,7,15].”

4. **Also the duration of first and second stage should be eventually evaluated in association to the use of oxytocin.**

All pregnant women received oxytocin

5. **I noted with interest a higher number of cases of prolonged deceleration in control group, please specify if they were in the context of epidural and eventually report the rate of epidural in the two groups.**

More cases of prolonged decelerations were observed in non-acidemia group, however, not reach statistically significant. No pregnant women who underwent epidural anesthesia were found in both groups. (In our center, epidural anesthesia did not routinely implement for routine intrapartum care)

____________________________________________________________________________________

Reviewer #2: Thank you very much for the opportunity to review this manuscript. The manuscript covers an important topic and is well written and structured. My comments and suggestions are as follows:

M + M:
**1. The term “perinatal asphyxia” is very vague and should be avoided in favor of Hypoxic-Ischemic Encephalopathy (HIE)Why did the authors exclude infants with no umbilical cord gas results? The diagnosis of “perinatal asphyxia” may be made even in their absence. The definition of “perinatal asphyxia” used in the manuscript is not congruent with the reference given (15) and, in particular, does not reflect current clinical practice. The definition of “perinatal asphyxia” should be revised as it requires biochemical criteria (usually a pH of less than 7.00 in the first hour of life) and neurologic criteria (usually encephalopathy or seizures).**

**Infants without neurological abnormalities and a cord blood pH anywhere < 7.20 would be classified as having perinatal acidosis.**

1. The authors changed the term of “perinatal asphyxia” to “perinatal acidemia” as the review’s suggestion.
2. Birth asphyxia or cerebral palsy may occur in antepartum or intrapartum period. Distal risk factors (genetic abnormalities, environmental/sociodemographic factors, and some placental abnormalities) often exert a pathogenic effect on fetal brain starting at conception or antepartum period. While, proximal risk factors (such as abruptio placenta, TTTS, chorioamnionitis) often exert a pathogenic effect on fetal brain at intrapartum period. The authors focused only at intrapartum period. Perinatal acidemia implied that fetuses had intrapartum hypoxic component. However, in revised version, the authors changed the term of asphyxia to acidemia, as the reviewer’s suggestion.

**1) How did the authors determine their sample size is “large enough”?**

The authors explained additional information about the required study population size.

In revised version: Materials and method (Page 4, line 75-80)

“The required study population size was determined based on comparison of area under the curve (AUC) of receiver operating characteristic (ROC) curves between the NICHD 3-tier system alone (estimated at 0.55) and the NICHD 3-tier system combined with risk factors or the FRI (estimated at 0.70) with approximately 12% of neonates having acidemia. For power of 80% and alpha of 0.05, and allowing for up to 20% incompleted data, 658 were required [16]”

**2) Why do the authors feel that single center design is a strength?**

**3) The limitations of this study should be elaborated in more detail, e.g. regarding generalizability.**

The authors edited the strength and limitations.

In revised version: Discussion (Page 11, line 198-206):

“Our study demonstrated that prediction of perinatal acidemia can be improved when combining risk factors with intrapartum FHR tracings. Our study could identify significant risk factors, which combined with NICHD 3-tier categories I or II improved detection of perinatal acidemia. The sample size was adequate to demonstrate significant risk factors, however, the study was conducted at a single tertiary center, which might reduce the generalizability of the findings. A future prospective studies will need to include a larger sample size for better reproducibility in identifying clinical risk factors, and include more abnormal FHR tracings, especially NICHD 3-tier category III cases. In summary, intrapartum EFM according to the NICHD 3-tier FHR classification system combined with selected clinical risk factors can improve the predictive ability for perinatal acidemia in neonates, and it is easy to use in general practice.”
