# Supplementary material for: Comparison of the predictive ability for perinatal acidemia in neonates between the NICHD 3-tier FHR system combined with clinical risk factors and the fetal reserve index
Source: PLoS One. 2022 Oct 20;17(10):e0276451. doi: 10.1371/journal.pone.0276451 (PMC9584503; doi:10.1371/journal.pone.0276451)
Supplement: Attachment — Submitted filename: Response to Reviewer.docx [file pone.0276451.s002.docx]

**Review Comments to the Author**

Please use the space provided to explain your answers to the questions above. You may also include additional comments for the author, including concerns about dual publication, research ethics, or publication ethics. (Please upload your review as an attachment if it exceeds 20,000 characters)

Reviewer #1: Some points need attention

Methods : definition of pathological deceleration

Pathologic deceleration was variable, late, prolonged deceleration.

Number of contraction 8 in 15 minutes to define tachysystole? the definition is different from NICHD classification

The authors have further explained about the contraction definition.

In revised version: Lines 102–103; “For contractions, abnormal uterine activity was defined as > 8 contractions in 20 minutes based on the FRI recommendations [9].”

Answers to my questions 4 and 5
Why was oxytocin used in all cases if arrest disorders were diagnosed only in a small part of the series? Does the authors refer to oxytocin for other reasons? or oxytocin is routinely used during labor? in both cases specify .

- If pregnant women did not have good uterine contraction spontaneously, oxytocin was routinely used in active phase of intrapartum for all cases.
- The reason for oxytocin usage was only for augmentation of labor for good uterine contraction in all cases.

The information is relevant for the readers
no cases with epidural? also this information is very relevant and should be added in the results section because the effect of epidural on fetal wellbeing is controversial

No cases with epidural anesthesia were found in both groups because in our center, epidural anesthesia did not routinely implement for routine intrapartum care (because of the limitation of anesthesiologist’s services). The authors have inserted this information to the results. “Results (line 118-199); No cases with epidural anesthesia were found in both groups.”

table
We need more data on the composition of samples. the We suggest the authors to perform a similar analysis eventually including a more "stringent" acidemic group

1. mean or median pH and BE of both groups

2. how many cases were defined as acidemic for pH, for BE or for both

The authors additionally described this information in the Results and Table 1

In revised version: Line 115-117; “Eighty neonates (11.8%) had evidence of perinatal acidemia. Of these, 60 neonates had pH ≤ 7.2, 47 neonates had base deficit ≥ 12mmoL/L, and 27 neonates had both pH ≤ 7.2 and BE ≥ 12 mmol/L.”

Table 1

|  | Acidemia  (n = 80) | Non-acidemia  (n = 594) | *P* |
| --- | --- | --- | --- |
| Advanced maternal age | 15 (18.8) | 122 (20.5) | 0.82 |
| Gestational age at delivery (weeks) (IQR) | 39 (38,40) | 39 (38,40) | 0.14 |
| BMI ≥ 30 (kg/m^2^) | 4 (5) | 32 (5.4) | 0.76 |
| Prior cesarean section | 2 (2.5) | 42 (7.1) | 0.19 |
| Preeclampsia | 4 (5) | 12 (2) | 0.11 |
| Fetal growth restriction | 0 | 3 (0.5) | 1 |
| Macrosomia | 1 (1.2) | 3 (0.5) | 0.39 |
| Oligohydramnios | 3 (1.8) | 8 (1.3) | 0.13 |
| Polyhydramnios | 0 | 1 (0.2) | 1 |
| Rupture of membrane | 69 (86.2) | 475 (80) | 0.23 |
| Arrest disorders of labor | 11 (13.8) | 48 (8.1) | 0.14 |
| Meconium passage | 18 (22.5) | 63 (10.6) | 0.004 |
| Chorioamnionitis | 3 (3.8) | 5 (0.8) | 0.05 |
| Abnormal second stage of labor | 2 (2.5) | 12 (2) | 0.67 |
| Neonatal outcomes |  |  |  |
| Median birth weight (IQR) | 3138 (2892,3409) | 3072 (2841,3390) | 0.41 |
| Median umbilical pH (IQR) | 7.17 (7.12,7.21) | 7.32 (7.29,7.36) | < 0.001 |
| Median umbilical base deficit  (mmol/L) (IQR) | 12.35 (9.90,14.40) | 5.60  (3.80,7.60) | < 0.001 |
| Umbilical pH ≤ 7.2 | 60 (75) | 0 | < 0.001 |
| Umbilical base deficit ≥ 12 mmol/L | 47 (58.7) | 0 | < 0.001 |
| 1-minute Apgar score < 7 | 36 (45) | 24 (4) | < 0.001 |
| Intensive neonatal resuscitation | 23 (28.7) | 15 (2.5) | < 0.001 |
| Intubation | 3 (3.8) | 5 (0.8) | < 0.001 |
| NICU admission | 4 (5) | 9 (1.5) | < 0.001 |

Note: The previous manuscript version, the authors made an incorrect symbol of pH. Actually, in the methods and analysis, acidemia was define as pH ≤ 7.2. The authors defined this definition according to reference [4] that the cord gas pH level above 7.2, it is unlikely that peripartum played a role in causing neonatal encephalopathy. So the revised version, the authors have carefully revised this point.

Reviewer #2: The authors have carefully revised the manuscript and have addressed all my comments. I have no further comments.
