# Supplementary material for: Comparison of the predictive ability for perinatal acidemia in neonates between the NICHD 3-tier FHR system combined with clinical risk factors and the fetal reserve index
Source: PLoS One. 2022 Oct 20;17(10):e0276451. doi: 10.1371/journal.pone.0276451 (PMC9584503; doi:10.1371/journal.pone.0276451)
Supplement: Attachment — Submitted filename: Response to Reviewers.docx [file pone.0276451.s003.docx]

Review Comments to the Author

Reviewer #1:

1. please verify the definition of pathological deceleration. You stated that the definition included all variable deceleration but this seems not clinically correct and in fact in the table the % of variable deceleration was higher than the % of pathological deceleration. Be sure to be consistent and clinically convinced of your conclusions

Response: We apologize for incorrect percentage of variable deceleration of Table 2. All information of Table 2 has now been checked and verified (Page 8, line 136). We also have checked the accuracy of the information for Table 1 and 3.
